# Evaluating Differential Metabolic Profiles by Prostate Cancer Risk Among Prostate Cancer Patients

**DOI:** 10.3390/metabo15120757

**Published:** 2025-11-21

**Authors:** Tuo Liu, Jahnvi Roorkeewal, Melissa A. Furlong, Shawn C. Beitel, Jefferey L. Burgess, Benjamin R. Lee, Juan Chipollini, Justin M. Snider, Ken Batai

**Affiliations:** 1 Department of Community, Environment, and Policy, Mel and Enid Zuckerman College of Public Health, University of Arizona, Tucson, AZ 85724, USA; tuoooliu@arizona.edu (T.L.); mfurlong@arizona.edu (M.A.F.); sbeitel@arizona.edu (S.C.B.); jburgess@arizona.edu (J.L.B.); 2 School of Nutritional Sciences and Wellness, University of Arizona, Tucson, AZ 85721, USA; jahnviroorkeewal@arizona.edu; 3 Department of Urology, College of Medicine-Tucson, University of Arizona, Tucson, AZ 85724, USA; brlee@arizona.edu (B.R.L.); jchipollini@arizona.edu (J.C.); 4 Department of Cancer Prevention & Control, Roswell Park Comprehensive Cancer Center, Buffalo, NY 14203, USA

**Keywords:** prostate cancer (PCa), urine metabolomics, PCa risk

## Abstract

**Background:** Currently there are no clinically validated biomarkers recommended for prostate cancer (PCa) risk stratification other than prostate-specific antigen (PSA). **Objective:** This study aimed to identify urine metabolites that are associated with the presence of high-grade PCa at the time of radical prostatectomy. **Methods:** Urine samples were collected from patients who underwent radical prostatectomy. High-resolution metabolomics were implemented using liquid chromatography mass spectrometry (LC-MS). To enhance metabolic feature identification, sample extracts were analyzed in two modes, C18 chromatography [reverse-phase (RP)] and hydrophilic interaction chromatography (HILIC). **Results:** This analysis included a total of 22 patients with PCa (10 high-grade and 12 low-grade) and identified 52 differential metabolites, 40 in RP and 12 in HILIC, at the *p*-value 0.05 level. Among these, methyl alpha-aspartyl phenylalaninate was most significantly differentiated, while 3-methylbutanoicacid had the largest difference (slope −3.488). In the pathway analysis, the histidine metabolism pathway was significantly enriched (*p* < 0.05) with an enrichment factor of 3.5. Although not statistically significant, alterations were also observed in the vitamin B12, B7 (biotin), B6, and B3 (niacin) pathways. **Conclusions:** These findings suggest that urinary metabolites may have the potential to differentiate high-grade from low-grade PCa. Our study also highlights the metabolic reprogramming that occurs as PCa becomes more aggressive and potential differences in dietary patterns.

## 1. Introduction

Prostate cancer (PCa) is the second most common cancer in men and the fourth most common cause of death worldwide [1]. In the United States (U.S.), approximately one out of every eight men will be diagnosed with PCa [2]. PCa is more common among older males (age > 50 years), African Americans, and men with family history of PCa. PCa grows slowly, and no treatment may be necessary when PCa is diagnosed at an early stage [3]. Digital rectal examination and serum prostate-specific antigen (PSA) are primary screening tests, but PSA testing among asymptomatic men can result in false positives and subsequently unnecessary prostate biopsy [4,5]. Aggressiveness and risk of progression is assessed based on PSA levels, stage at presentation, and grade group. Treatments for PCa are dependent on the risk groups (e.g., active surveillance for low risk and radical prostatectomy or radiation therapy for intermediate and high risk PCa), and a combination of therapy may be necessary for fast growing and high risk PCa [6,7].

Currently, there are some biomarkers, such as urine PCA3, HOXC6, and DLX1 mRNA, as well as ERG and PCA3 exosomal RNA, for risk stratification that are for clinical use, but there are no clinically validated biomarkers used routinely other than PSA pre-diagnostically to identify aggressive PCa. Commercially available molecular biomarkers using tissue samples for prostate biopsy or radical prostatectomy, such as Prolaris, Decipher, and Oncotype Dx, are also shown to be useful for identifying aggressive PCa and predicting disease progression [8]. Blood and urine biomarkers are non-invasive techniques, and when used combined with clinical information, these biomarkers can improve risk stratification and predict PCa progression [9,10]. However, routine use of these molecular biomarkers as well as blood and urine biomarkers are not recommended because of the lack of, or insufficient, data from prospective studies and data showing that the use of biomarkers improve long-term outcomes [8,11]. Low-circulating tumor DNA and cost are limiting factors for the use of circulating tumor DNA for risk stratification [12,13]. While these biomarkers have great potential for reducing overdiagnosis and overtreatment, better non-invasive and cost-efficient biomarkers are necessary for risk stratification as well as monitoring disease progression.

In such a case, metabolomics can be a very useful tool. Metabolites are a necessary part of every essential biochemical reaction in the human body. They can be essential biomarkers that could provide ameliorative and sensitive indication of biological systems [14]. This can constitute metabolomic patterns representing changes in initiation and progression of PCa. Urine metabolomics is a potential non-invasive strategy that can help identify key biomarkers for identifying clinically significant PCa and enhance PCa care [15]. Urine is easy to procure and handle, and it has a lower protein content and consists of various metabolites [16]. However, many previous urine metabolomics studies focused on identifying metabolites that differentiate PCa from benign prostate hyperplasia (BPH) or control, and only a few studies investigated the urine metabolic biomarkers for clinically significant or aggressive PCa.

The goal of the current exploratory study was to identify urinary biomarkers that are associated with high-grade PCa (≥8) in patients with localized PCa who underwent radical prostatectomy.

## 2. Materials and Methods

### 2.1. Study Population and Sample Collection

Patients with localized PCa who underwent radical prostatectomy at Banner University Medical Center, Tucson between 2018 and 2020 were eligible for this study. Urine samples were collected from patients intraoperatively, at the time of radical prostatectomy, with a Foley catheter under sterile conditions. All patients were fasting prior to undergoing surgery. Urine samples were aliquoted and stored immediately after the surgery at −80 °C in the University of Arizona Cancer Center (UACC) Biorepository. Patients’ demographic, clinical, and pathologic data were corrected by the UACC Biorepository personnel. The data collected by the Department of Urology team were de-identified and linked to samples and additional data from the UACC. Aggressiveness of PCa was defined based on grade group categorized into low (<7), intermediate (7), and high (≥8), with the high-grade group being aggressive. The study protocol was approved by the University of Arizona Institutional Review Board.

### 2.2. Sample Preparation

Urine samples were prepared by spiking 10 μL of ^13^C labeled internal standard mix (which consisted of a 20 μM mix of L-Phenylalanine (D8, 98%), Taurocholic Acid (D4, 98%), DL-Valine (D8, 98%), Succinic Acid (13C4, 99%; 2,2,3,3-D4, 98%), covering both negative and positive ionization for quality control only) in a 1:1 solution of urine and ice-cold acidified methanol to provide an acidified solution at 0.1% formic acid. The resulting mixtures were vortexed, centrifuged, and the supernatants were collected. Samples were extracted in randomized batches of 48 samples, which included a batch QC of standardized non-patient urine. The sample extracts were then analyzed.

### 2.3. High-Resolution Metabolomics

We performed high-resolution metabolomics (HRM) according to the established methods of Najdekr et al. [17]. Analyses were performed on a Thermo Scientific Exploris Orbitrap 480 (Thermo Scientific, Waltham, MA, USA) with randomized samples. To enhance metabolic feature identification, sample extracts were analyzed in two modes using a dual-column, dual-polarity approach including a C18 chromatography (reverse-phase, RP) with positive electrospray ionization (ESI+), along with a hydrophilic interaction (HILIC) chromatography with negative ESI−.

Following a 1 μL sample injection, RP separation was accomplished using a 1.8 µm, 2.1 × 150 mm HSS T3 Column (ACQUITY Premier HSS T3 Column, Waters, Milford, MA, USA) and methanol gradient (A = 99.9% water 0.1% formic acid, B = 99.9% Methanol 0.1% formic acid) consisting of an initial 3 min period of 99% A and 1% B, followed by linear increase to 50% B at 11 min and then increase to 95% B and a hold for 2 min.

Separation by HILIC was accomplished using a 1.7 µm, 2.1 mm × 150 mm Amide column (Waters ACQUITY Premier BEH Amide Column, Waters, Milford, MA, USA) with 10 mM ammonium formate and acetonitrile gradient (A = 10% water, 90% ACN, 10 mM ammonium formate and 0.1% formic acid, B = 50% water, 50% ACN, 10 mM ammonium formate and 0.1% formic acid) consisting of an initial 3 min period of 99% A and 1% B, followed by linear increase to 50% B at 11 min and then increase to 95% B and a hold for 2 min.

The Mobile phase flow rate was 0.3 mL/min for both the RP and HILIC methods. The mass spectrometer was operated using ESI mode at a resolving power of 60,000 and mass-to-charge ratio (m/z) range 65–1000 Da. High-resolution detection of m/z features was accomplished by a maximum injection time of 100 ms and custom AGC target (Normalized AGC Target (%)) of 50%.

To evaluate systematic variation, lab blanks were run immediately before AcquireX samples at the beginning of each batch. A pooled quality control (QC) sample was run after every 30 injections and later were used in Compound Discoverer to filter features based on a RSD > 30% filtration, with QC samples clustered tightly within the sample cluster in PCA analysis. Two other quality control sample types were also prepared: the internal laboratory sample extracted with every batch, along with a pooled sample consisting of an aliquot of all extracts from the extraction batch.

### 2.4. Metabolite Annotation and Statistical Analysis

Metabolite annotation was accomplished using the Compound Discoverer Software (version 3.3, Thermo Scientific). To safeguard annotation quality, a mass tolerance of 5 ppm was applied. Mass spectra were annotated against several libraries. We first annotated features against an in-house library built on standards (>300 metabolites) purchased from MetaSci, Inc. (Toronto, ON, CA). We also utilized online libraries, including MzCloud, ChemSpider, Masslist, and Metabolika, to capture signals outside the in-house library. The Schymanski index was used to showcase annotation confidence for each metabolite (Supplemental Table S1).

Metabolic features were uniquely defined by their mass-to-charge ratio (*m*/*z*), retention time, and ion intensity (or relative abundance, hereafter, ion intensity). In reverse-phase (RP), 22% of metabolites were robustly detected (missing in < 25% of samples), while 25% of metabolites were sparsely detected (missing in > 75% of samples); in HILIC, 8% of metabolites were robustly detected (missing in < 25% of samples), while a large proportion (72%) was sparsely detected (missing in > 75% of samples). Based on the assumption of a mixed-missingness mechanism, missing values on ion intensity were imputed using random forest algorithms as implemented in the Compound Discoverer Software (Thermo Scientific, Waltham, MA, USA). Normalization was carried out to remove unwanted variation including batch effects and hydration factor by applying a linear regression model that adjusts for specific gravity factor for urine sample and batch number. All metabolic features’ ion intensities were then prepared by log2 transformation for further analysis. The outcome of normalization was inspected by plotting the total intensity against samples. There was no significant remaining batch effect after normalization (Supplemental Figure S1).

A multiple regression model, with log-transformed ion intensity being the response and PCa grade group being the primary predictor (high- vs. low-grade), was fitted for every urinary feature while adjusting for important covariates. Linear Model ANOVA and Fisher’s Exact Test for Count Data were applied for two-group comparison on continuous and categorical demographic variables, respectively, to identify important covariates for PCa grade. Significant covariates were then included in the main model. The coefficient for the primary predictor was interpreted as the log2 fold change Log2(Y2/Y1)=slope×Grade Group. We conducted a complete-case analysis and excluded observations with missing demographic values from the model. We defined differential metabolites as those with a *p*-value smaller than 0.05. We adjusted for multiple testing by controlling the family-wise error rate at false discovery rate (FDR) 0.05 level. A volcano plot was made for each separation-ESI mode to demonstrate the overall differential status. All statistical analyses were performed in the R programming environment (version 4.3.0).

### 2.5. Pathway Analysis

Following normalization and statistical analysis, we performed pathway analysis to investigate the difference in metabolic profiles by PCa grade at the biological level. All identified metabolic features were included as the reference set in the pathway analysis using Mummichog (version 2) as implemented in the MetaboAnalyst (version 5.0). The KEGG library was used as the knowledge base. A list of differential hits was included as the sample set. To reduce false positive matches, we restricted all annotated metabolites to be present in primary ion mode corresponding to the ESI modes, and only pathways with a size of at least 3 (contains more than 3 metabolites) were included in the output. Pathway analysis was performed using Fisher’s exact test to evaluate the over-representation of the mapped metabolites in each pathway. We used the Benjamini–Hochberg procedure to control the false discovery rate (FDR) across tested pathways. Pathways were considered statistically significant at FDR *q* < 0.05. Uncorrected (raw) *p*-values from Fisher’s exact test are reported for completeness and as exploratory evidence; we explicitly note when results meet only the uncorrected *p* < 0.05 threshold but do not meet the FDR criterion.

## 3. Results

### 3.1. Study Population and Sample

This analysis included a total of 22 patients (10 high-grade and 12 low-grade PCa). Upon checking on the distributions of demographic variables by PCa grade group, age (years) and BMI (kg/m^2^) present significantly different distributions between the low- and high-grade groups. The high-grade group was older, with a mean age of 71.5 years, and heavier, with a BMI of 30.5, than the low-grade group with a mean age of 63.0 years and BMI of 27.1 (Table 1). We also examined the differences in Hispanic ethnicity, hypertension, diabetic and marital status, insurance category, and cancer history, and found no significant difference between the high- and low-grade groups.

### 3.2. High-Resolution Metabolomics

High-resolution mass spectrometry (HMR) was employed to perform untargeted metabolomics profiling on urine samples collected from PCa patients. The samples in this study are a sub-set of a larger metabolomics study and therefore were extracted in six separate batches and run as a single batch, with each sample processed using both hydrophilic interaction liquid chromatography (HILIC) and reverse-phase (RP) chromatography run sequentially to maximize metabolite coverage. To ensure consistency and account for instrumental drift, a pooled quality control (QC) sample—composited from all study groups—was injected every 30 injections throughout the run. Metabolite intensities were normalized to the signal from these QC injections, enabling reliable comparison across all runs. Following normalization, the data demonstrated good consistency across extraction batches, facilitating downstream statistical analysis.

High-resolution metabolomics identified 643 and 1723 urinary features in HILIC(−) and RP(+) mode, respectively, by annotating against a combination of in-house and online libraries. After removing features with more than 25% missing values in our samples, a total of 539 and 1662 urinary features were retained for HILIC(−) and RP(+) mode, respectively. All matched metabolites from both modes were included in the differential analysis and subsequent pathway analysis.

### 3.3. Differential Analysis and Statistical Analysis

**Figure 1:**
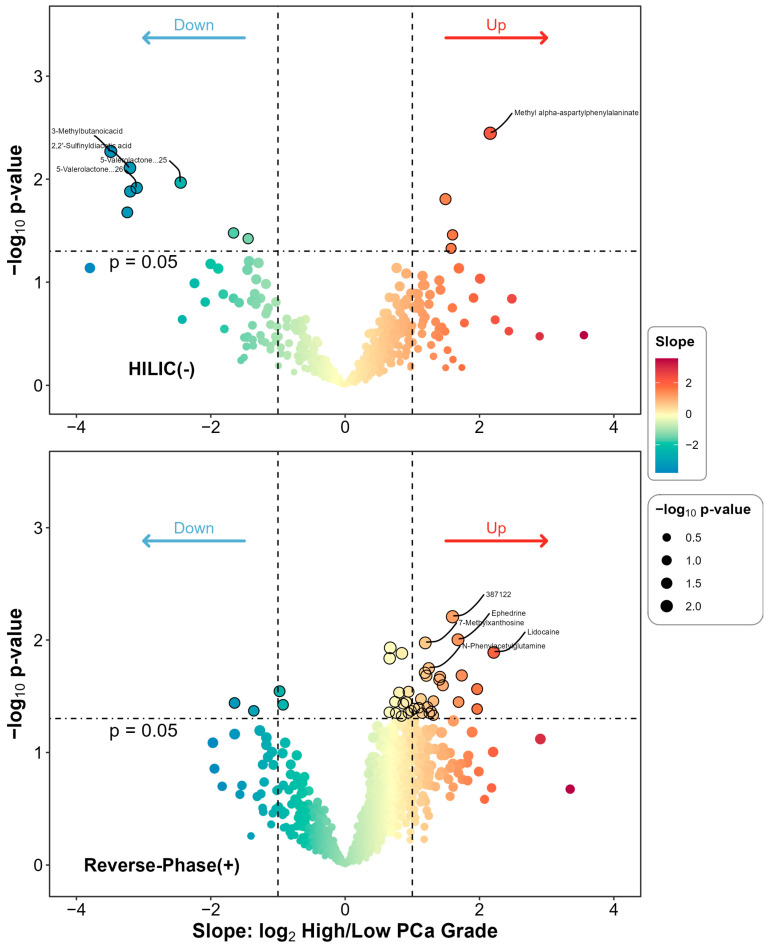
Differential metabolites comparing high- to low-grade group among PCa patients. The subplots are for HILIC(−) and RP(+) mode, respectively. For each metabolite detected in each mode, a covariate-adjusted linear regression model was fitted. The slope term was derived from the main model with the main predictor being binary PCa grade (High vs. Low) and the response being log2 transformed metabolite’s relative abundance, while adjusting for important covariates including age and BMI. The statistical significance was also derived from the main model and log10 transformed into the figure.

After adjustment for covariates that were significantly different by PCa grade group, including age and BMI, we identified 12 (HILIC) and 40 (RP) differential metabolites at a *p*-value < 0.05 level. However, none were statistically significant at the FDR 0.05 level. The list of differential metabolites is given and ranked by *p*-value in ascending order in Table 2. Figure 1 presents the overall as well as individual differential status for the urinary features included in the differential analysis. Overall, there were 52 metabolites, 12 in HILIC(−) and 40 in RP(+) mode, identified. Among these, methyl alpha-aspartyl phenylalaninate was most significantly differentiated with the smallest *p*-value (*p* = 0.004), while 3-methylbutanoicacid had the largest difference (slope −3.488). A complete list of metabolites, along with their slopes, *p*-values and FDR corrected q-values, can be found in Supplemental Tables S1 and S2.

### 3.4. Pathway Analysis

**Figure 2:**
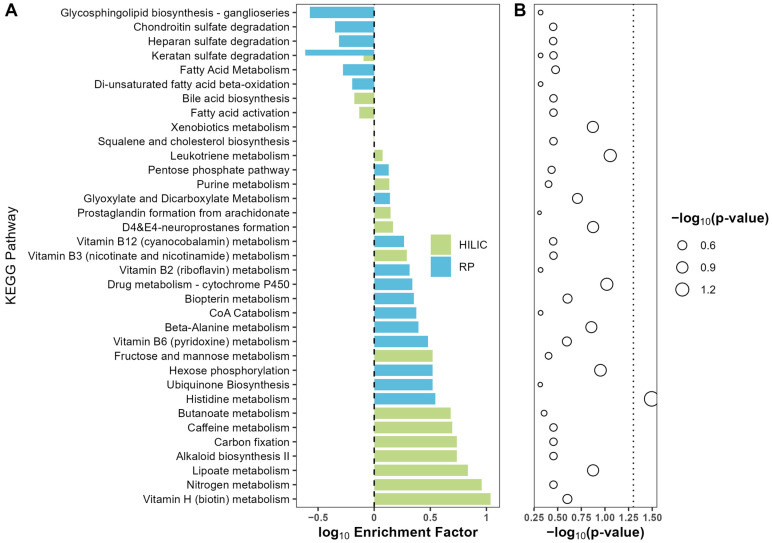
Enrichment plot for the comparison between high- vs. low-grade group. HILIC and RP enriched pathways were indicated by color green and blue, respectively. Differential metabolites were used as input for enrichment analysis with MetaboAnalyst 5.0. For each metabolic pathway, an over-representation analysis was performed to derive statistical significance for enrichment. (**A**) Enrichment factor was calculated as the ratio of the number of matched metabolites and the expected number of metabolites for a given pathway and presented at log10 scale. (**B**) The *p*-values were calculated by Fisher’s exact test for over-representation analysis in each pathway. Statistical significance was transformed into −log10(*p*-value) scale and indicated by the size of circles, with large circle size indicating higher significance.

A total of 44 and 70 pathways were reported back from pathway analysis in HILIC(−) and RP(+) mode, respectively. Metabolic pathways with a *p*-value no greater than 0.5 and containing at least three metabolites were included in Figure 2. There were 18 pathways from each mode. Only the histidine metabolic pathway was significantly enriched at the *p*-value 0.05 level, with an enrichment factor of 3.5. However, after adjusting for multiple testing, no significantly enriched pathways were reported at FDR *q* = 0.05 level. Also, while not significant, alterations in vitamins B12, B7 (biotin), B6, and B3 (niacin) pathways were enriched.

**Figure 3:**
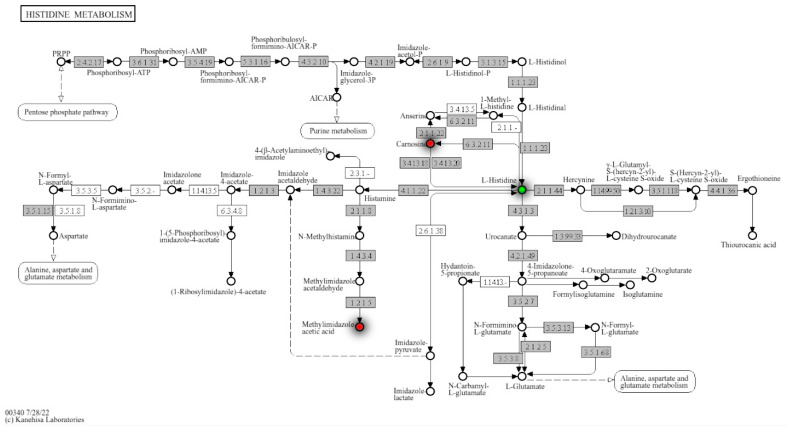
Histidine metabolism with detailed regulation status of matched metabolites from annotation and Mummichog. Mummichog outputs hits by its own annotation algorithms and the matches with our own annotation were mapped in the metabolism map, where red dots identify the upregulated metabolites in this specific pathway.

**Figure 4:**
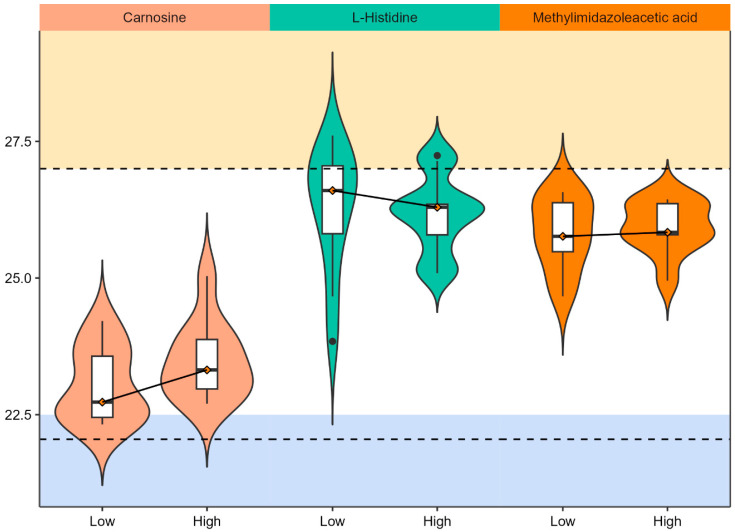
Violin plots for matched metabolites in histidine metabolism. The x-axis indicates PCa grade status while y-axis indicates the ion intensity after log-2 transformation. The solid line connecting the median values of ion intensity indicates the directionality of change by PCa grade. The boxplot consists of the minimal, first quartile, median, third quartile, and the maximum ion intensity of the metabolites. Two dashed lines are added arbitrarily to highlight the range of ion intensity that capture the most metabolites in the urine.

To further investigate histidine metabolism regulation in PCa, metabolite hits were mapped to KEGG pathways. Carnosine, L-histidine, and methylimidazole acetic acid were identified in histidine metabolism and detected at *p*-value ≤ 0.1 in RP mode (Figure 3). Carnosine and methylimidazole acetic acid were upregulated, and L-Histidine was downregulated in the high PCa grade group as compared to the low PCa grade group (Figure 4). More enrichment analysis details can be found in Supplemental Table S2.

## 4. Discussion

There is a critical need for non-invasive biomarkers that can determine PCa status and disease trajectory and stratify patients based on their risk of disease progression. Metabolites in urine and blood can potentially be used as diagnostic and prognostic biomarkers. Our study highlights that the metabolic reprogramming occurs as PCa becomes more aggressive. Cancer cells reprogram their metabolism to meet proliferative demands and adapt to stressful microenvironments by increasing nutrient uptake and incorporating them into biosynthetic pathways, potentially leading to altered excretion of metabolites in urine. Here we observed alterations in very abundant urine metabolites including carnosine and L-histidine, major components in the histidine pathway [18]. Other enriched pathways included beta alanine and various vitamin metabolic pathways, all of which have precedence in the literature with regard to PCa status [15,19,20]. Taken together, the profile of dysregulated metabolites given here could represent a signature for evaluating PCa status in patient populations.

With the onset of cancer and as cancer progresses, mutations in oncogenes and tumor suppressor genes alter signaling and metabolic pathways, which lead to metabolic rewriting. Metabolites are the end product of various biochemical processes, and the differences in the metabolites reflect the disease process altering the tumor microenvironment. In PCa and many other cancer types, the tricarboxylic acid (TCA) cycle is often altered as the tumor cells rely on glucose for energy production. Amino acids are involved in tumor pathogenesis and progression as fuel for tumor cells and signaling molecules [21]. Amino acid metabolism also plays an important role in PCa progression driven by Androgen Receptor signaling [22], and previous metabolomics studies have demonstrated that amino acid metabolites are dysregulated in urine from PCa patients, including patients with aggressive PCa [14,23,24,25].

Here we observed significant alterations in histidine metabolism as well as enrichment of beta-alanine metabolites. Histidine is an interesting metabolite that is downregulated in the serum and urine from PCa patients compared to BPH patients and controls [26,27]. Other metabolites in the histidine metabolic pathway are also downregulated in PCa (e.g., methylhistidine and acetylhistidine in Fernandez-Peralbo 2016 [27] and 3-methyl-L-histidine in Huang 2023 [15]). On the other hand, metabolic profiling studies using prostate tissue specimens from radical prostatectomy have shown that histidine and 1-methyl-histidine were more abundant in tumors compared to non-tumor tissues [28,29,30]. A serum metabolomics study of the Alpha-Tocopherol, Beta-Carotene Cancer Prevention (ATBC) trial in Finland provides support for implicating histidine in aggressive and lethal PCa, demonstrating that *N*-acetylhistine is associated with increased risk of lethal PCa compared to controls [31] and that histidine is associated with increased risk of PCa mortality in PCa cases [32]. Histamine phosphorylation is crucial in cell cycle regulation, and histidine metabolism is dysregulated in aggressive and lethal PCa.

Our finding of decreased urinary histidine appears to contrast with previous reports of increased tissue histidine in PCa [28,29,30]. However, these observations are mechanistically consistent and reflect compartmentalized metabolism. PCa cells likely exhibit enhanced histidine uptake and retention, creating a ‘metabolic sink’ that depletes circulating pools and reduces urinary excretion. Supporting this interpretation, we observed concurrent upregulation of histidine-derived urinary metabolites—carnosine and methylimidazole acetic acid—indicating active histidine metabolism within tumor tissue with the excretion of downstream products. Thus, elevated tissue histidine and decreased urinary histidine represent complementary findings of cancer-associated metabolic reprogramming, where local tumor metabolism differs from whole-body balance reflected in urine

Beta-alanine and carnosine may also play some roles in aggressive PCa, but the exact relationship with PCa aggressiveness is not clear. Carnosine is a molecule consisting of beta-alanine and histidine and was observed as a significant molecule in our study. Carnosine is thought to have anti-tumor properties [33], and the plasma level of carnosine dipeptidase 1 is reduced in aggressive PCa [34]. Elevated levels in high-grade PCa patients in our study may suggest increased excretion of carnosine in patients with high-grade PCa. Contrary to our study showing increased carnosine levels in patients with high-grade PCa, Huang et al. found increased levels of beta-alanine in urine from the benign and indolent PCa patients compared with patients with aggressive PCa [15]. Differences in dietary patterns in the patient populations in two studies (the U.S. and Taiwan) may have contributed to the inconsistent findings. While produced endogenously, carnosine can also be a metabolite related to meat consumption, and in a systematic review by Nouri-Majd et al., it was linked to PCa risk, though, without dietary records, we cannot distinguish between dietary contribution and endogenous metabolic dysregulation [35]. Therefore, urine carnosine levels may reflect differences in dietary patterns or risk among grades.

Moreover, methyl alpha-aspartyl phenylalaninate was the most significantly differentiated metabolite. Phenylalaninate is an aromatic amino acid anion that is the conjugate base of phenylalanine, and increased circulating levels of phenylalanine are shown to be associated with higher risk of PCa mortality [32]. Phenylalanine, like methyl alpha-aspartyl phenylalinate, is also a metabolite of aspartame, which has had mixed and controversial effects on cancer and cancer grade [36,37,38], though again, a study including dietary records would be needed to establish any link. Taken together, amino acid metabolic pathways are dysregulated in aggressive PCa, resulting in differential secretion of these metabolites detected in urine. These metabolites may also point to differences in dietary habits that are associated with risk; however, the cross-sectional nature of the study and the lack of validating dietary evidence are limitations.

In this study, glycosphingolipids were not abundant in aggressive PCa, as they were negatively enriched, which supports a previous metabolomics study of urine extracellular vesicles that found reduced glycerophospolipids in PCa compared to BPH [39]. Previous plasma metabolomic profiling studies, on the other hand, have identified various types of lipids associated with PCa development, aggressiveness, progression, and mortality [20,21,22,23,24,25]. Glycerophospholipids (e.g., phosphocholine) and sphingolipids (e.g., ceramides and sphingomyelins) were some of the top predictive metabolites in these previous studies that were abundant in aggressive PCa. However, these studies yielded inconsistent findings of specific lipid metabolites, probably due to different study designs or behavioral factors of study populations, such as obesity and diet, influencing plasma metabolite abundance. Taken together, this suggests that aggressive PCa cells may sequester glycosphingolipids for tumor growth, reducing their urinary excretion and highlighting the need for further research into lipid metabolism’s role in PCa progression.

Although not statistically significant, metabolites in the lipoate metabolic pathway were also enriched. Lipoate or lipoic acid has antioxidant effects. It reduces cell growth, migration, and invasion, and induces apoptosis in PCa [40,41] and other cancer types [42,43]. Furthermore, a major enzyme in lipoate metabolism, lipoic acid synthetase (LIAS), has been suggested as a novel biomarker for prognosis and immune response in various cancers [44]. Taken together, this pathway has the potential as a target for cancer therapeutic agents, specifically due to its anti-metastatic properties.

In recent years, the growing interest in alternative medicines for health management has led to a rise in the consumption of vitamins, minerals, and other dietary supplements, especially among the general population and individuals diagnosed with cancer or other chronic diseases. Our research identified alterations in vitamins B12, B7 (biotin), B6, and B3 (niacin) pathways in patients with aggressive PCa compared to patients with low-grade PCa, consistent with metabolomics findings by Xu et al. [19], who identified biotin metabolism as enriched in serum from PCa compared to BPH. However, the relationship between B vitamins and PCa risk remains controversial. Johansson et al. [20] initially reported an association between circulating vitamin B12 levels and advanced-stage PCa in the European Prospective Investigation into Cancer and Nutrition (EPIC) study, but this finding was not replicated in the subsequent larger collaborative analysis by the Endogenous Hormones, Nutritional Biomarkers, and Prostate Cancer Collaborative Group, which found no significant association [45]. These discrepancies likely reflect differences in statistical power, heterogeneity across populations, and fundamentally different biological questions: prospective circulating B12 measurements address whether vitamin status influences cancer initiation, while metabolomic alterations observed by Xu et al. and in our study may reflect the metabolic consequences of established tumors rather than etiological risk factors.

The biological relevance of B-vitamin dysregulation centers on their role as coenzymes in one-carbon metabolism, which regulates DNA methylation, nucleotide biosynthesis, and cellular proliferation. Both B12 and B6 function as essential cofactors in the folate-mediated one-carbon pathway, while biotin serves as a cofactor for carboxylases in lipid metabolism, and niacin is a precursor for NAD+, critical for energy metabolism and DNA repair. Evidence from folate studies illustrates this complexity: the Aspirin/Folate Polyp Prevention Study found that folic acid supplementation increased PCa risk [46], and the Endogenous Hormones, Nutritional Biomarkers, and Prostate Cancer Collaborative Group demonstrated that circulating folate was associated with advanced-stage and high-grade PCa [45]. Conversely, dietary folate intake was linked to lower PCa risk in the same study, suggesting that supplemental and dietary forms may influence risk differently, potentially reflecting dose-dependent effects or differences in folate metabolism. Our observation of coordinate dysregulation across multiple B-vitamin pathways (B12, B6, B7, B3) in high-grade PCa suggests that aggressive tumors exhibit altered metabolic requirements for these interconnected pathways to support rapid proliferation and maintain redox homeostasis. This interpretation reconciles conflicting findings: circulating B-vitamin levels may have limited or context-dependent associations with cancer risk, while metabolic pathway alterations in established tumors reflect cancer-associated metabolic reprogramming. Although vitamins are crucial for physiological functions, the impact of vitamin deficiencies or excessive supplementation on PCa development and progression remains uncertain and likely depends on metabolic context, genetic background, and disease stage.

A similar metabolic profiling of urine from PCa patients performed by Chow et al. discovered four metabolites that produced a AUC of 0.842 for the ROC [47]. Among these were vitamin D and cytochrome p450 metabolites that demonstrated significant alterations between healthy controls and PCa groups. Interestingly, our study identified altered trends in the p450 pathway (Figure 2), indicating that both studies observed disruption in this pathway. The convergence of findings across independent cohorts suggests that cytochrome P450 dysregulation represents a robust metabolic signature of PCa rather than a study-specific artifact. This consensus is biologically significant given the multifaceted role of P450 enzymes in both steroid hormone metabolism and xenobiotic processing, two processes intimately linked to prostate carcinogenesis [48,49]. In the context of our study, the observed alterations in P450 metabolism may reflect disrupted androgen signaling and impaired vitamin D activation, both of which are established drivers of PCa progression [50]. The identification of cytochrome P450 metabolism as a marker of PCa may be confounded, as this enzyme family also metabolizes many drugs commonly administered to treat PCa or comorbidities, making it difficult to distinguish disease-related metabolic changes from those induced by therapy. However, other studies have suggested that EGF-driven dysregulation of CYP27B1 expression in prostate cells may contribute to PCa development and the use of vitamin D metabolites could be an effective treatment [51]. These findings may reinforce the significance of cytochrome P450-mediated vitamin D metabolism in PCa, suggesting that disruptions in this pathway may serve as both biomarkers and therapeutic targets for disease management.

There are some limitations in the current study. First, this study focused on low- and high-grade PCa cases at the time of radical prostatectomy. There was a small number of patients in the low- and high-grade groups, resulting in a small sample size, and we were able to identify only one significantly altered metabolic pathway. Due to the small sample size, this study serves an exploratory purpose and the generalizability of the results in this study is limited. Second, the samples lacked diversity, and many of them were non-Hispanic White patients. Future studies need to include patients from diverse backgrounds. We also leveraged an existing biobank, and clinical information was retrospectively collected. Important clinical variables, such as pre-treatment PSA and medication as well as information on factors influencing urine metabolites, such as dietary intake and dietary supplement use, were not available or collected. Metabolites identified or enriched metabolic pathways may have been due to the excretion of food that patients had prior to surgery, dietary supplements, or medications. The influence of these factors on urine metabolism should be considered in future studies. Furthermore, the exact time interval between urine collection and centrifugation for debris removal was not systematically documented. Delays beyond 30 min could potentially introduce pre-analytical variability through metabolite degradation or cell lysis, which should be considered when interpreting results. Finally, patients underwent radical prostatectomy at a single academic institution, and our findings may not be generalizable.

## 5. Conclusions

This study, which aimed to identify non-invasive biomarkers, identified some metabolites and enriched metabolic pathways in urine associated with aggressive PCa. These findings indicate metabolic reprogramming across multiple metabolic pathways, underscoring the complexity of this disease. Additional studies with large, diverse patient populations are necessary to develop non-invasive urine biomarkers to predict disease trajectory and stratify patients based on their risk of disease progression.

## Figures and Tables

**Table 1:** Summary statistics of demographics in PCa patients (N = 22) by grade group. ^1^ Linear Model ANOVA. ^2^ Fisher’s Exact Test for Count Data.

Variable	Low-Grade (N = 12)	High-Grade (N = 10)	*p*-Value
Age	63.00 (60.50, 68.00)	71.50 (68.75, 74.50)	0.002 ^1^
BMI			0.022 ^1^
(Missing)	0	3	
Median (Q1, Q3)	27.05 (24.88, 27.90)	30.50 (27.15, 32.00)	
Ethnicity			0.571 ^2^
Hispanic	1 (8.3%)	2 (20.0%)	
Non-Hispanic	11 (91.7%)	8 (80.0%)	
pT_Stage			0.004 ^2^
2	9 (75.0%)	1 (10.0%)	
3	3 (25.0%)	9 (90.0%)	
Hypertension			1.000 ^2^
No	7 (58.3%)	6 (60.0%)	
Yes	5 (41.7%)	4 (40.0%)	
Diabetes			0.571 ^2^
No	11 (91.7%)	8 (80.0%)	
Yes	1 (8.3%)	2 (20.0%)	
Marital Status			0.330 ^2^
Divorced	2 (16.7%)	0 (0.0%)	
Married	10 (83.3%)	8 (80.0%)	
Single	0 (0.0%)	1 (10.0%)	
Widowed	0 (0.0%)	1 (10.0%)	
Insurance			0.204 ^2^
(Missing)	2	1	
Medicaid	2 (20.0%)	1 (11.1%)	
Medicare	3 (30.0%)	7 (77.8%)	
Other_Public	1 (10.0%)	0 (0.0%)	
Private	4 (40.0%)	1 (11.1%)	
Family Prostate Cancer History			0.333 ^2^
(Missing)	1	4	
No	6 (54.5%)	5 (83.3%)	
Yes	5 (45.5%)	1 (16.7%)	
Other Cancer History			1.000 ^2^
(Missing)	1	4	
No	9 (81.8%)	5 (83.3%)	
Yes	2 (18.2%)	1 (16.7%)	

**Table 2:** Top differential metabolites with high annotation confidence comparing participants with high vs. low PCa grade. Positive slopes indicated elevated levels in the high-grade group while negative slopes indicated the opposite.

Mode	Analytes	Slope	*p*-Value
HILIC	Methyl alpha-aspartyl phenylalaninate	2.160	0.004
HILIC	3-Methylbutanoicacid	−3.488	0.005
HILIC	2,2’-Sulfinyldiacetic acid	−3.204	0.008
HILIC	5-Valerolactone25	−2.448	0.011
HILIC	5-Valerolactone26	−3.103	0.012
HILIC	3-Methylbut−2-enal	−3.199	0.013
HILIC	(1-Ribosylimidazole)-4-acetate	1.494	0.016
HILIC	(2E)-3-Methyl-4-(sulfooxy)-2-butenoic acid	−3.243	0.021
HILIC	5-Valerolactone266	−1.662	0.033
HILIC	Dihydrolipoamide	1.599	0.035
HILIC	1-deoxynojirimycin	−1.444	0.038
HILIC	2-Hydroxy-4-oxo-1,2,4-butanetricarboxylic acid	1.577	0.047
Reverse-Phase	387122	1.599	0.006
Reverse-Phase	Ephedrine	1.680	0.010
Reverse-Phase	7-Methylxanthosine	1.192	0.011
Reverse-Phase	HMBA	0.671	0.012
Reverse-Phase	Lidocaine	2.212	0.013
Reverse-Phase	Choline	0.844	0.013
Reverse-Phase	Euxanthone	0.663	0.015
Reverse-Phase	N-Phenylacetylglutamine	1.245	0.018
Reverse-Phase	3,5-Dimethoxybenzoic acid	1.188	0.020
Reverse-Phase	2-([4-(Benzyloxy) anilino] carbonyl amino)-N-(3-morpholinopropyl) benzamide548	1.735	0.021
Reverse-Phase	Glucuronamide739	1.209	0.021
Reverse-Phase	7-Deoxyloganate	1.412	0.021
Reverse-Phase	L-gamma-Glutamyl-L-leucine823	1.402	0.023
Reverse-Phase	1-Methylhistidine	1.455	0.025
Reverse-Phase	1-(beta-D-Ribofuranosyl)-1,3,4,7-tetrahydro-2H-1,3-diazepin-2-one	1.965	0.027
Reverse-Phase	gamma-Glutamyl-gamma-aminobutyraldehyde451	-0.976	0.029
Reverse-Phase	3-Methylcrotonylglycine812	0.944	0.029
Reverse-Phase	Pregabalin33	0.803	0.029
Reverse-Phase	Cantharidin1336	1.128	0.034
Reverse-Phase	2,6-Toluene diisocyanate704	1.314	0.035
Reverse-Phase	4-(4-methylphenyl)-2-phenyl-1,3-thiazole881	0.742	0.035
Reverse-Phase	5-Hydroxy-6-methoxyindoleglucuronide524	0.911	0.036
Reverse-Phase	Gramine1180	1.688	0.036
Reverse-Phase	3,5-dibenzylpyridine-2,6-diamine84	−1.646	0.036
Reverse-Phase	8-Amino-7-oxononanoate1541	0.866	0.037
Reverse-Phase	3-Hydroxy-N-[(3S)-2-oxotetrahydro-3-furanyl]decanamide642	−0.924	0.038
Reverse-Phase	Volkenin	1.225	0.039
Reverse-Phase	2-Oxooctadecanoicacid	1.096	0.040
Reverse-Phase	NP-017152	1.031	0.041
Reverse-Phase	D-Iditol	1.965	0.041
Reverse-Phase	Fusarin C	−1.360	0.043
Reverse-Phase	3-Methoxytyramine	0.981	0.043
Reverse-Phase	1-(alpha-D-Glucopyranosyluronosyl)-3-[(2S)-1-methyl-5-oxo-2-pyrrolidinyl] pyridinium	1.282	0.043
Reverse-Phase	Flumequine	0.657	0.044
Reverse-Phase	Promacyl 887	0.941	0.045
Reverse-Phase	3-Hydroxy-3-[(3-methylbutanoyl) oxy]-4-(trimethylammonio)butanoate…1418	0.753	0.045
Reverse-Phase	Ranitidine S-oxide	1.146	0.045
Reverse-Phase	Supinine41	1.252	0.045
Reverse-Phase	di(3-pyridyl) 5-(tert-butyl) isophthalate	1.313	0.047
Reverse-Phase	3-Morpholino-4-tetrahydro-1H-pyrrol-1-ylcyclobut-3-ene-1,2-dione	0.838	0.048

## Data Availability

The data presented in this study are available on request from the corresponding author. The data are not publicly available due to privacy or ethical restrictions.

## Supplementary Materials

The following supporting information can be downloaded at: https://www.mdpi.com/article/10.3390/metabo15120757/s1. Table S1: A complete list of metabolites with model output, including slopes, *p*-values, and FDR corrected q-values, together with the Schymanski index. Table S2: Details of enrichment analysis, organized by separation-ESI mode. Figure S1: Batch effect plot for HILIC(−) and RP(+) mode. Samples in either red or blue were run in the same batch. The urine sample index was mapped to x-axis, and the y-axis was sample total intensity after normalization.
