# Supplementary material for: Evaluating Differential Metabolic Profiles by Prostate Cancer Risk Among Prostate Cancer Patients
Source: Metabolites. 2025 Nov 21;15(12):757. doi: 10.3390/metabo15120757 (PMC12735200; doi:10.3390/metabo15120757)
Supplement: Supplementary file 1 [file metabolites-15-00757-s001.zip › metabolites-3902306-supplementary.pdf]

Evaluating Differential Metabolic Profiles by Prostate Cancer Risk among Prostate Cancer Patients

Tuo Liu[1], Jahnvi Roorkeewal[2], Melissa A. Furlong[1], Shawn C. Beitel[1], Jefferey L. Burgess[1], Benjamin R Lee[3], Juan Chipollini[3], *Justin M. Snider[2], *Ken Batai[4]

[1]Department of Community, Environment, and Policy, Mel and Enid Zuckerman College of Public Health, University of Arizona, Tucson, US
[2]School of Nutritional Sciences and Wellness, University of Arizona, Tucson, US
[4]Department of Urology, College of Medicine-Tucson, University of Arizona, Tucson, AZ, US
[5]Department of Cancer Prevention & Control, Roswell Park Comprehensive Cancer Center, Buffalo, NY, US

*Corresponding authors Ken.Batai@roswellpark.org, Elm & Carlton St. Buffalo NY 14263 & Justinsnider@arizona.edu , 1230 N Cherry Ave. Tucson AZ 85721

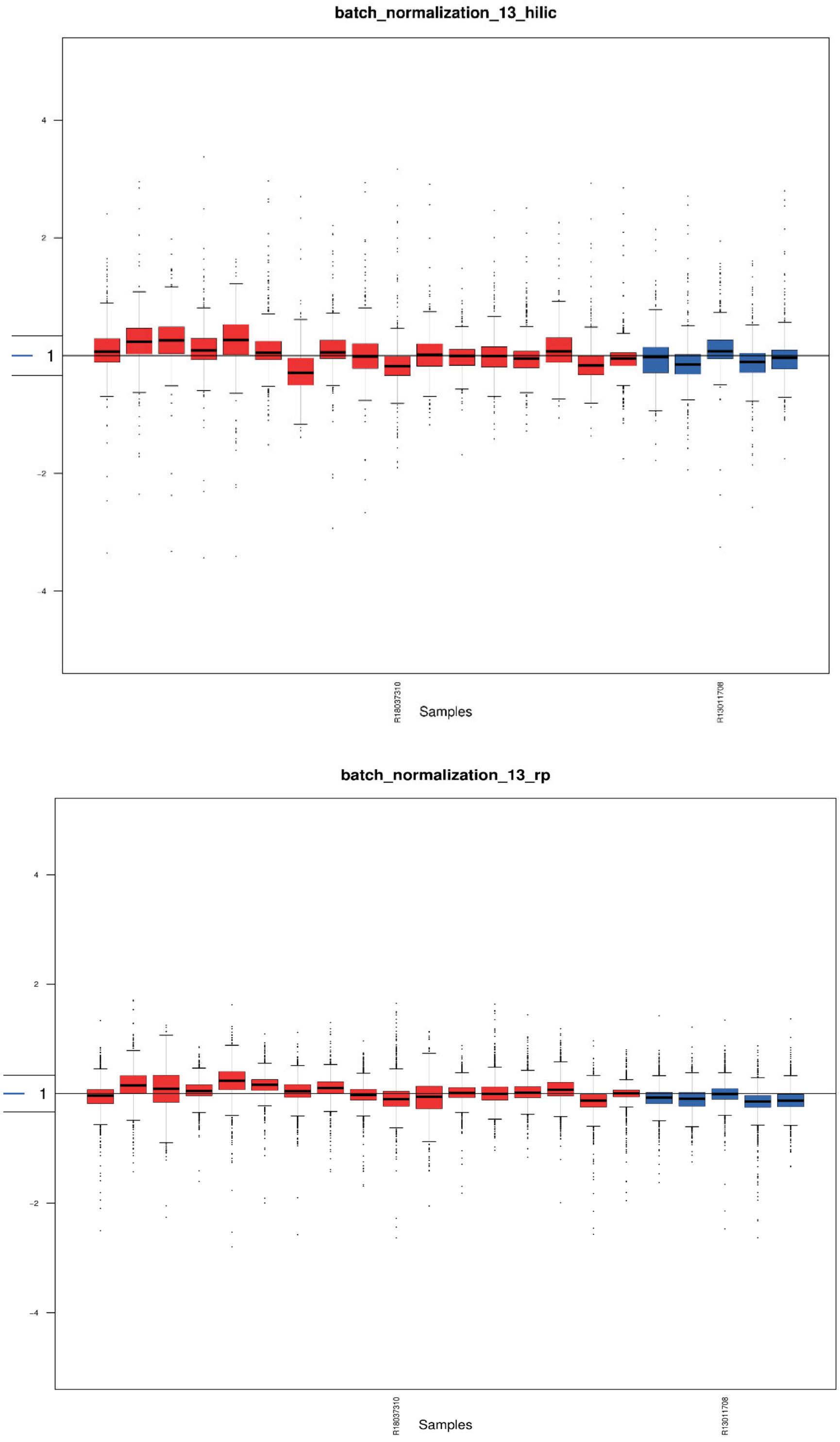

Supplemental Figure S1. Batch effect plot for HILIC(-) and RP(+) mode. Samples in either red or blue were run in the same batch. The urine sample index was mapped to x-axis, and the y-axis was sample total intensity after normalization.

After log-transformation, the ion intensities of the urinary features were roughly the same magnitude, which allowed for apple-to-apple comparison among urinary features. Although within-batch variation existed, there was no clear batch-wise variation observed from the above plots, after normalization.
